# Nutrients or processing? An analysis of food and drink items from the UK National Diet and Nutrition Survey based on nutrient content, the NOVA classification and front of package traffic light labelling

**DOI:** 10.1017/S0007114524000096

**Published:** 2024-05-14

**Authors:** Samuel J. Dicken, Rachel L. Batterham, Adrian Brown

**Affiliations:** 1 Centre for Obesity Research, Department of Medicine, University College London (UCL), London WC1E 6JF, UK; 2 National Institute for Health Research, Biomedical Research Centre, University College London Hospital (UCLH), London W1T 7DN, UK; 3 Bariatric Centre for Weight Management and Metabolic Surgery, University College London Hospital (UCLH), London NW1 2BU, UK

**Keywords:** Front of package labelling, Ultra-processed food, NOVA classification

## Abstract

UK front of package labelling (FOPL) informs consumers on the nutrient content of food. However, FOPL does not consider food processing, and with the UK government being urged to act on ultra-processed food (UPF), whether UPF should be added to FOPL is unclear. This study compared food and drink in the UK National Diet and Nutrition Survey (NDNS) Intake24 database based on FOPL, nutrient content and NOVA classification, to understand whether UPF are covered by dietary recommendations for foods high in fat, salt and sugar. NDNS items were coded into minimally processed food (MPF), processed culinary ingredients, processed food and UPF according to the NOVA classification and FOPL traffic lights. UPF contained greater energy, fat, saturated fat (SF), total sugar (TS) and salt than MPF. UPF had a greater odds of containing red FOPL and an unhealthier overall FOPL score (OR:4·59 (95 % CI: 3·79, 5·57); OR:7·0 (95 % CI: 6·1, 8·2), respectively) and lower odds of containing green FOPL (OR:0·05 (95 % CI: 0·03, 0·10)), compared with MPFs. For items with no red FOPL, UPF still contained greater energy, fat, SF, TS and salt than MPF. However, several UPF have healthier FOPL scores. UPF had an unhealthier nutritional profile and FOPL score than MPF. For items with no red FOPL, UPF still had an unhealthier profile than MPF, with a higher energy density. Importantly, not all UPF were unhealthy according to FOPL. These results indicate partial overlap between FOPL, nutrient content and NOVA classification of UK food and drink products, with implications for UK food and drink labelling.

Diets high in saturated fat, added sugar and salt (HFSS) are associated with increased risks of mortality and non-communicable disease^(1–3)^. As such, UK dietary guidelines recommend that the public reduces their intake of these nutrients, to lower the risk of developing non-communicable diseases such as obesity, type 2 diabetes and CVD and all-cause mortality^(4,5)^.

Nutritional guidance is communicated to the public through multiple strategies including the Eatwell Guide, and front of package labelling (FOPL), which is used to help guide consumer choice at the point of purchase^(6,7)^. FOPL systems differ across countries, from simple non-interpretive nutrient information to interpretive semi-directive colour coded nutrient information (e.g. multiple traffic light (MTL) system in the UK), to interpretive directive advice to support consumer choices (e.g. Nutri-Score in Europe)^(8)^. Current FOPL focus on the energy and nutrient content of products. Compared with no label, FOPL systems help consumers to better rank the healthiness of food products^(9)^. In the UK, the Eatwell Guide advice is provided through the semi-directive MTL system, which assigns a green, amber or red colour on a FOPL based on whether the content of fat, saturated fat, salt or sugar is low, medium or high, respectively^(6)^.

Besides nutrient content, mounting evidence shows that processing may also impact on health. A number of food processing classifications exist. The NOVA classification is the most common definition used in the academic literature and was determined by the UK Scientific Advisory Committee on Nutrition as being the only classification to be potentially applicable to the UK population, meeting all five of their screening criteria^(10)^. The NOVA classification categorises food and drink into four groups: minimally processed food, processed culinary ingredients, processed food and ultra-processed food^(11)^. Of particular interest are ultra-processed foods, which are industrially reformulated products, typically with five or more ingredients resulting in highly palatable, long lasting, readily accessible, cheap products^(12)^. Ultra-processed foods now constitutes a significant proportion of adult daily energy intake^(13)^, with nearly 60 % of intake in UK adults^(14)^. Such a large intake from ultra-processed food is of concern, due to the fact that greater ultra-processed food consumption is associated with increased risks of non-communicable disease and all-cause mortality^(15,16)^.

Indeed, diets high in ultra-processed food tend to display poorer nutritional profiles, being higher in fat, saturated fat, free sugar and lower in fibre, protein and micronutrients^(13)^. However, evidence suggests that the associations between ultra-processed food and negative health outcomes appear to be independent of the nutrient content of the diet, or the overall diet pattern^(15,16)^. In the Italian Moli-Sani cohort, both lower dietary nutritional quality (measured as Nutri-score) and greater ultra-processed food intake were independently associated with increased risks of cardiovascular and all-cause mortality^(17)^. Further joint analyses showed an attenuated effect of Nutri-Score with mortality, but not of ultra-processed food^(17)^. Such findings indicate that dietary nutritional quality and the extent and purpose of processing may capture different, distinct components/aspects of diet on health. However, the recent Scientific Advisory Committee on Nutrition report on processed foods and health^(10)^ suggested that (ultra-) processing may be covered by existing UK dietary recommendations in terms of HFSS foods. Therefore, there is a need for understanding whether (ultra-) processing matters and whether it can be used to guide consumer purchasing behaviour.

A number of potential mechanisms have been suggested to explain the adverse impacts of a diet high in ultra-processed food, relating to their typically poorer nutritional quality and aspects of their processing. In particular, ultra-processed foods may be more energy dense^(18)^ and have hyperpalatable properties that may encourage increased energy intake^(16,19,20)^, which may be linked to their negative impact on health. The lower protein content of an ultra-processed diet has also been suggested to be a factor driving excess consumption^(16,21)^.

Studies outside of the UK have compared the NOVA classification with nutrient indices such as the Nutrient Rich Food Index^(22)^, FOPL tools such as Nutri-Score or MTL or nutrient profiling models such as those by the WHO^(23–28)^. Such studies find an inverse, partial association between ultra-processed food intake and dietary quality, or typically poorer FOPL profiling in ultra-processed foods compared with minimally processed foods, but also find that not all ultra-processed foods are nutritionally inferior. One UK study indicated that processed and ultra-processed foods tend to have a poorer nutrient profile compared with minimally processed food^(12)^. No study, however, has examined in detail how the nutrient content of a nationally representative sample of foods and drinks in the UK and their FOPL MTL score varies, based on the NOVA classification. In addition, the overlap between indices of hyper-palatability and the NOVA classification has not been fully determined^(20)^, nor has the energy density profile of UK food and drinks across the NOVA classification. This is important to determine whether ultra-processed foods are adequately captured by existing HFSS dietary recommendations and FOPL MTL and to understand and whether food processing groups differ nutritionally in the UK. The aim of this study was to examine the association between the extent and purpose of processing of UK food and drinks with their nutrient content. The objective was to assess how the NOVA classification maps onto the UK food and drink supply in the National Diet and Nutrition Survey (NDNS) Rolling Programme Year 12 database, by assessing the overlap with FOPL MTL scoring and nutritional characteristics.

## Methods

### Data sources

NDNS is a repeated cross-sectional survey, providing detailed dietary intake from a nationally representative sample of the UK population, aged 1·5 years and older and living in private households, since 2008^(29)^. Years 1–11 (2008/9 to 2018/19) of the survey were assessed using food records across four consecutive days. In Year 12 (2019 to 2020), four-day food records were replaced with four non-consecutive, multiple-pass, 24-h recalls as the dietary assessment method in the NDNS survey^(30)^. Recalls were conducted using Intake24, a web-based, automated, self-administered 24-h dietary recall tool (https://intake24.co.uk)^(31)^. Food and drink item names and subgroups used in Intake24 were obtained from the Intake24 team. The corresponding NDNS nutrient databank with the most up-to-date public nutrient information for each food item was obtained from the UK Data Service (https://beta.ukdataservice.ac.uk). Further details on Intake24 and NDNS Year 12 have been published elsewhere^(30)^.

### NOVA classification

Food and drink items were coded according to the NOVA classification^(11)^ (see online Supplementary Materials for full detail of the coding process including coding of homemade dishes). Years 1–11 of NDNS included a nutrient database with mixed dishes separated into their constituent ingredients (e.g. lettuce, mushrooms and mayonnaise from a salad mixed dish), which has been previously coded^(32)^. In NDNS Year 12, the food database was updated by removing redundant items, and the nutrient databank was updated to reflect the most current food composition data^(30)^. Food items were no longer disaggregated. For example, the nutrient databank contains sandwiches and salads, rather than the individual components of sandwiches and salads, as in previous NDNS assessments.

Each food and drink item in the Year 12 dataset was individually coded. NOVA coding of items was conducted with the authors blind to the Intake24 nutrient database. The coding process was discussed between sd and a Registered Senior Specialist Dietitian (AB), and a description of the process is provided in the online supplementary materials. Classification was determined based on the NOVA classification definitions^(11)^, item name, subgroup code, best representation from products available in leading UK supermarkets and the NOVA group of the corresponding item in the NDNS Years 1–11 database from previous publications^(32)^. Initial coding was conducted by sd. Both authors agreed on the classification for ambiguous food items and food groups. Where it was unspecified or ambiguous as to whether a food item was home-made or ready-made, the authors agreed on the most appropriate classification based on the most likely method of obtaining or preparing the food, by reflecting on the range of, and ingredients within, corresponding products sold from leading UK supermarkets.

### Front of package label classification

The NDNS nutrient databank was coded into FOPL MTL according to the Department of Health and Food Standards Agency guidance for fat, saturated fat, total sugar and salt content^(6)^. Items with low content for a given nutrient are coded green, moderate content as amber and high content as red. The nutrient cut-offs are provided in Table 1. To allow for comparability, items were coded per 100 g of food or drink. As per FOPL guidance, drink items included lower cut-offs for amber or red colour coding per 100 g of drink, which was assumed equivalent to 100 ml of drink.

**Table 1.** FSA FOPL cut-off points for fat, saturated fat, total sugar and salt content. The colours do not represent claims. Green denotes ‘low’, amber ‘medium and red ‘high’ amounts of that nutrient in a food/drink

Nutrient	Green FOPL traffic light	Amber FOPL traffic light	Red FOPL traffic light
Fat /100 g: Food	≤ 3·0 g	> 3·0 g to ≤ 17·5 g	> 17·5 g
Fat /100 ml: Drink	≤ 1·5 ml	> 1·5 ml to ≤ 8·75 ml	> 8·75 ml
Saturated fat /100 g: Food	≤ 1·5 g	> 1·5 g to ≤ 5·0 g	> 5·0 g
Saturated fat /100 ml: Drink	≤ 0·75 ml	> 0·75 ml to ≤ 2·5 ml	> 2·5 ml
Total sugar /100 g: Food	≤ 5·0 g	> 5·0 g to ≤ 22·5 g	> 22·5 g
Total sugar /100 ml: Drink	≤ 2·5 ml	> 2·5 ml to ≤ 11·25 ml	> 11·25 ml
Salt /100 g: Food	≤ 0·3 g	> 0·3 g to ≤ 1·5 g	> 1·5 g
Salt /100 ml: Drink	≤ 0·3 ml	> 0·3 ml to ≤ 0·75 ml	> 0·75 ml

FOPL, front of package label.

### Statistical analysis

Normally distributed variables were described using means and confidence intervals, and variables that were not normally distributed were described using medians and interquartile range (IQR). Categorical variables were described using counts and percentages. Comparisons of non-parametrically distributed nutrient variables between NOVA groups were analysed using Kruskal–Wallis analysis of variance, with Bonferroni correction for multiple comparisons. Categorical variables were analysed using *χ* ^2^ tests.

The number of products within each NOVA group, the average nutrient and energy content per 100 g and distribution of FOPL traffic lights were described. The average nutrient content per 100 g and the distribution of individual traffic lights for each nutrient was then compared between NOVA groups. FOPL traffic lights for all four nutrients (fat, saturated fat, total sugar and salt) combined (i.e. MTL) were then compared. The presence of any red or green FOPL traffic lights (*v*. no red or green FOPL traffic lights), the number of red FOPL traffic lights and the number of green FOPL traffic lights (none, one, two, three or four) and the distribution of the overall FOPL MTL score (an 8-level categorical measure ranging from four green to four red FOPL traffic lights (four reds, three reds and one amber were combined due to few items in these categories)) was then compared across NOVA groups. Due to small numbers of items across categorical levels, processed foods and processed culinary ingredients were grouped for the ordinal analyses and for the binary green *v*. no green FOPL traffic light analysis.

Unadjusted regression analyses were then conducted to examine the relationship between NOVA groups as the categorical independent variable and FOPL traffic lights or nutrient content as the dependent variables. Binary regression was used to analyse the odds of containing at least one red traffic light (*v*. no red traffic light) and the odds of containing at least one green traffic light (*v*. no green traffic light). Ordinal logistic regression was used to model the odds of containing a higher number of red traffic lights, the odds of containing a higher number of green traffic lights and to model the odds of having a better overall FOPL MTL score.

Based on previous evidence suggesting that UK consumers more readily avoid red traffic lights over choosing green traffic lights when identifying healthier products^(33,34)^, items were stratified as ‘healthy’ or ‘unhealthy’ by the presence or absence of a red FOPL traffic light across the four nutrients. Subgroup analyses then compared the nutrient and energy content of products with healthier *v*. less healthy FOPL MTL, first within ultra-processed foods (i.e. healthy *v*. unhealthy products), and then between NOVA food groups (i.e. healthy products across NOVA groups). Comparisons between ultra-processed foods with or without a red FOPL traffic light were analysed using Mann–Whitney *U* tests.

To consider the wider nutritional characteristics of food items proposed as potential mechanisms of ultra-processed foods in the NDNS database, items were characterised by previously defined quantifiable definitions of hyper-palatable foods (HPF) based on a systematic review of descriptive definitions of hyper-palatability^(35)^. Items were classified into three clusters, containing: (1) fat and Na (> 25 % kcal from fat, ≥ 0·30 % Na content by weight); (2) fat and simple sugars (> 20 % kcal from fat, > 20 % kcal from naturally occurring or added sugars) and (3) carbohydrates and Na (> 40 % kcal from carbohydrates, ≥ 0·20 % Na by weight)^(35)^. The proportions of HPF were then compared across NOVA groups. Drinks items were excluded from the HPF analysis, as the definition is only applicable to food items.

Items outside of the NOVA classification were removed prior to analysis (e.g. fish oil supplements and multivitamins).

### Sensitivity analysis

The nutrient and energy content of items across NOVA groups were analysed with a binary regression modelling the odds of containing above average nutrient content across the database (i.e. an above median *v*. median or below nutrient content. Linear regression was also used to determine the association between NOVA group and the number of red or green FOPL traffic lights. The full FOPL MTL score analysis was repeated using linear regression (where a green FOPL traffic light scored 1, amber scored 2 and red scored 3, for a combined continuous score ranging from 4 (four greens) to 12 (four reds)) and repeated using binary regression to model the odds of an above average (above median *v*. median or below) overall FOPL MTL score.

In subgroup analyses, comparisons of nutrient and energy content between subgroups with or without a red FOPL traffic light were repeated with a binary regression, to model the odds of an above average nutrient or energy content (above median *v*. median or below value). Subgroup analyses were also repeated by further stratifying products based on the presence of two or more green and no red FOPL traffic lights. Statistical significance was set at a *P* value < 0·05. Data were analysed in SPSS V29.0.

## Results

The Intake24 dataset contained 3105 items; 109 items were designated outside of the NOVA classification (e.g. fish oil supplements and multivitamins). When aligned with the NDNS nutrient databank, a further sixteen did not contain a number corresponding to a respective item in the latest version of the databank, leaving a total of 2980 items in the final analysis. Over half of the food and drink items were ultra-processed foods (*n* 1650, 55·4 %), around a third of the items were minimally processed foods (*n* 986, 33·1 %), 9·5 % were processed foods (*n* 283) and 2·0 % (*n* 61) were processed culinary ingredients.

**Fig. 1.**
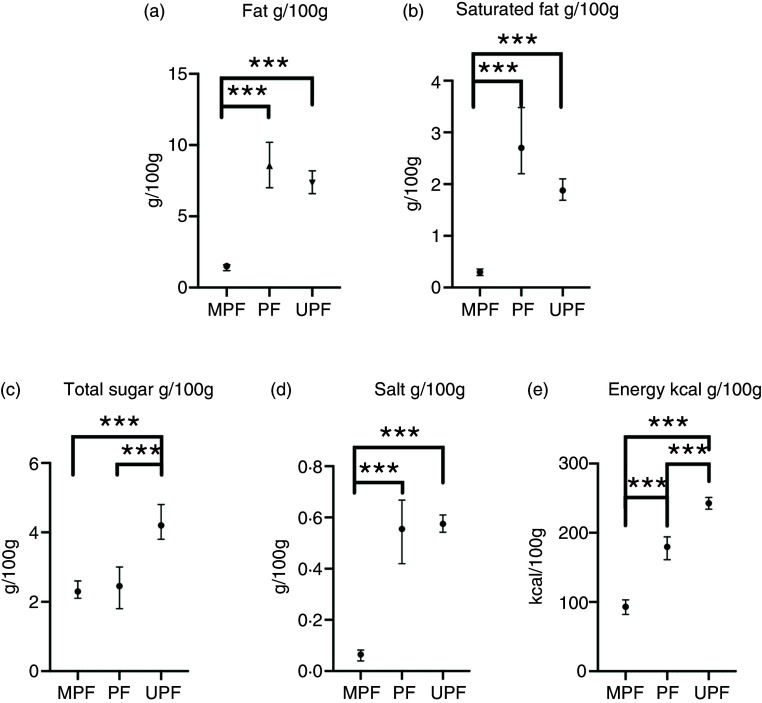
Average fat (a), saturated fat (b), total sugar (c), salt (d) and energy (e) content across NOVA food groups (*n* 2980). Median with 95 % CI. ***denotes significance at *P* < 0·001 conducted from Kruskal–Wallis ANOVA with Bonferroni correction for multiple comparisons. MPF, minimally processed food; PF, processed food; UPF, ultra-processed food.

Table 2 presents the average nutrient and energy content of all items, and within each NOVA group. The median content of fat, saturated fat, total sugar, salt and energy per 100 g was 5·1 g (IQR: 0·8, 13·5), 1·3 g (IQR: 0·2, 4·2), 3·2 g (IQR: 1·1, 11·1), 0·3 g (IQR: 0·05, 0·86) and 181·0 kcal (IQR: 77·0, 320·0), respectively. Minimally processed foods had significantly lower average fat, saturated fat and energy content per 100 g than other NOVA groups (all *P* < 0·001) (Fig. 1(a)–(e)). Ultra-processed foods contained significantly more fat, saturated fat, total sugar, salt and energy per 100 g than minimally processed foods (all *P* < 0·001). Ultra-processed foods had significantly greater average total sugar content per 100 g than other NOVA groups (all *P* < 0·001). Processed culinary ingredients had significantly greater energy content per 100 g than other NOVA groups (*v*. ultra-processed foods: *P* = 0·009, *v*. minimally processed foods or processed culinary ingredients: *P* < 0·001). Processed foods contained significantly more fat, saturated fat, salt and energy per 100 g than minimally processed foods (all *P* < 0·001), but a similar amount of total sugar (*P* = 0·167). The fat, saturated fat and salt content of processed foods did not differ to ultra-processed foods, but the energy density was significantly lower (*P* < 0·001). Processed culinary ingredients tended to have the highest average fat and saturated fat per 100 g of all NOVA groups, but was not significantly different from ultra-processed foods. Sensitivity analysis with binary regression showed similar findings (online Supplementary Table 1). Ultra-processed foods contained a similar amount of protein as minimally processed foods and processed foods, and a similar quantity of fibre as minimally processed foods, but more fibre than processed foods (*P* < 0·001), but this was not meaningfully different. Ultra-processed foods also had a significantly lower water content than minimally processed foods (75·9 g/100 g (IQR: 63·0, 87·7) *v*. 49·3 g/100 g (IQR: 16·1, 72·7), *P* < 0·001) (online Supplementary Table 2).

**Table 2.** Fat, saturated fat, total sugar, salt and energy content per 100 g by NOVA group

Nutrient	Total (*n* 2980)		MPF (*n* 986)		PCI (*n* 61)		PF (*n* 283)		UPF (*n* 1650)		*P*-value
	Median	IQR	Median	IQR	Median	IQR	Median	IQR	Median	IQR	
Fat (g/100 g)	5·1	0·8, 13·5	1·5 (a)	0·3, 6·5	19·1 (b)	0·0, 98·3	8·4 (b)	0·3, 16·4	7·4 (b)	2·1, 15·8	< 0·001
Saturated fat (g/100 g)	1·3	0·2, 4·2	0·3 (a)	0·07, 1·9	12·0 (b)	0·0, 32·5	2·7 (b)	0·1, 5·9	1·9 (b)	0·6, 5·7	< 0·001
Total sugar (g/100 g)	3·2	1·1, 11·1	2·3 (ab)	0·4, 5·1	0·0 (a)	0·0, 2·9	2·5 (b)	0·7, 9·7	4·2 I	1·7, 18·9	< 0·001
Salt (g/100 g)	0·27	0·05, 0·86	0·07 (a)	0·01, 0·2	0·03 (a)	0·0, 0·12	0·56 (b)	0·11, 1·05	0·58 (b)	0·18, 1·07	< 0·001
Energy (kcal/100 g)	181·0	77, 320	94·0 (a)	36·0, 180·3	378·0 (b)	181·0, 885·0	178·0 (c)	84·0, 290·0	243·0 (d)	121·8, 371·0	< 0·001

IQR, inter-quartile range; MPF, minimally processed food; PCI, processed culinary ingredient; PF, processed food; UPF, ultra-processed food.

Unlike letters indicate significantly different *P* < 0.05.

Pairwise comparisons conducted using Kruskal–Wallis ANOVA with Bonferroni correction for multiple comparisons.

### Traffic light labelling

**Fig. 2.**
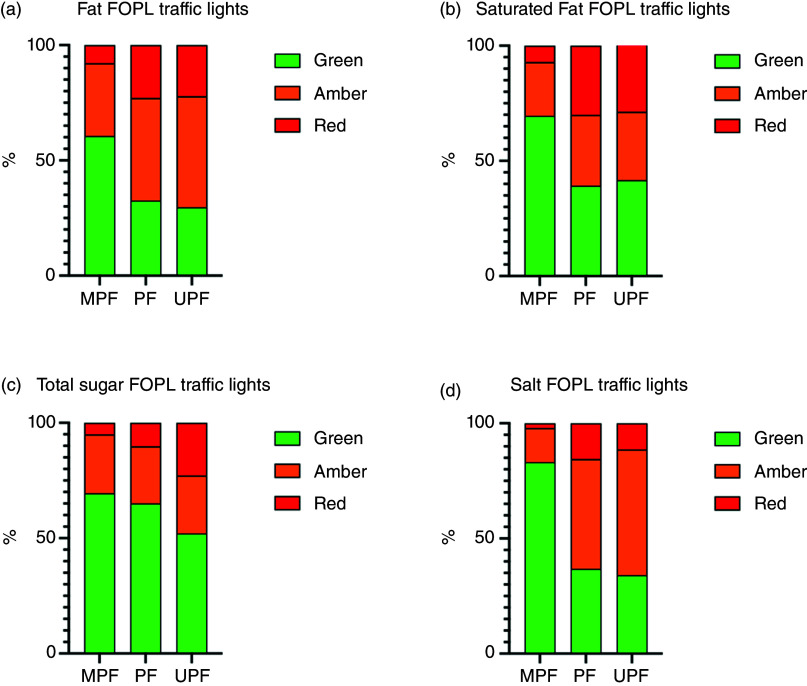
Percentage of red, amber, and green FOPL traffic lights across fat (a), saturated fat (b), total sugar (c) and salt (d) by NOVA group (processed culinary ingredients not shown). MPF *n* 986; PF *n* 283; UPF *n* 1650. FOPL, front of package label; MPF, minimally processed food; PF, processed food; UPF, ultra-processed food.

Items were then coded according to FOPL traffic lights. The total number of red FOPL traffic lights was 18·3 % (*n* 545) for fat, 22·3 % (*n* 665) for saturated fat, 15·7 % (*n* 467) for total sugar and 8·7 % (*n* 259) for salt (online Supplementary Table 3). The number of green FOPL in the database was 40·4 % (*n* 1203) for fat, 50·6 % (*n* 1509) for saturated fat, 59·5 % (*n* 1773) for total sugar and 51·5 % (*n* 1534) for salt. Figure 2(a)–(d) presents the percentage of red, amber and green FOPL traffic lights across fat, saturated fat, total sugar and salt, by NOVA group (processed culinary ingredients not shown). The proportions of green, amber and red FOPL traffic lights for fat, saturated fat, total sugar, salt significantly differed across NOVA groups (all *P* < 0·001). Minimally processed foods had a greater proportion of green FOPL traffic lights and a lower proportion of red FOPL traffic lights, whereas ultra-processed foods had a lower proportion of green FOPL traffic light, and a higher proportion of red FOPL traffic light.

### Combined front of package label traffic lights

When considering the presence of any red or green FOPL traffic lights per item, approximately two-thirds of items contained no red FOPL traffic lights (*n* 1846, 61·9 %), whereas only 9·1 % (*n* 270) of items contained no green FOPL traffic light (online Supplementary Table 4). Stratifying by NOVA group, the proportions of items with no red or green FOPL traffic lights significantly differed (*P* < 0·001). Most minimally processed foods contained no red FOPL traffic lights (83·2 %; 820 out of 986) with only 16·8 % containing one or more red FOPL traffic lights, compared with 51·8 % of ultra-processed foods containing no red FOPL traffic lights (855 out of 1650), and nearly half with at least one red FOPL traffic light (48·2 %; 795 out of 1650). Examples of minimally processed products that contain one or more red FOPL traffic lights include nuts, seeds, dried fruit, whole milk, eggs and some cuts of red meat. Less than 1 % of all minimally processed foods contained no green FOPL traffic light for fat, saturated fat, total sugar or salt (eight out of 986), compared with 14·0 % of ultra-processed foods (231 out of 1650). Examples of minimally processed products that contain no green FOPL traffic lights include mixed spice, five spice and dried milk. 99·2 % of minimally processed foods contained at least one green traffic light, compared with 86·0 % of ultra-processed foods. In binary regression analyses (Table 3), ultra-processed foods had a higher odds of containing one or more red FOPL traffic lights compared with minimally processed foods (OR: 4·59 (95 % CI: 3·79, 5·57)), as did processed foods (OR: 3·69 (95 % CI: 2·77, 4·92) and processed culinary ingredients (OR: 28·54 (95 % CI: 13·80, 59·05)) (*P* < 0·001). Similarly, ultra-processed foods had a lower odds of containing one or more green FOPL traffic light compared with minimally processed foods (OR: 0·05 (95 % CI 0·03, 0·10)), as did processed culinary ingredients and processed foods (combined, OR: 0·08 (95 % CI 0·04, 0·18) *P* < 0·001).

**Table 3.** Binary regression modelling the association between NOVA group and the presence of one or more red/green FOPL traffic lights *v*. No red/green FOPL traffic lights

	Exp(Beta)	95 % confidence interval	*P* value
		Lower, Upper	
One or more red *v*. no red FOPL traffic lights			
UPF	4·593	3·788, 5·569	< 0·001
PF	3·690	2·765, 4·924	< 0·001
PCI	28·541	13·795, 59·047	< 0·001
One or more green *v*. no green FOPL traffic lights			
UPF	0·050	0·025, 0·102	< 0·001
PF and PCI	0·083	0·038, 0·182	< 0·001

FOPL, front of package label; UPF, ultra-processed food; PF, processed food; PCI, processed culinary ingredient; MPF, minimally processed food.

Reference = MPF.

Higher score indicates greater odds of having red or green FOPL traffic lights vs. no red or green FOPL traffic lights.

### Ordinal front of package label multiple traffic light score

When considering the number of items with at least one red FOPL traffic light (*n* 1134, 38·1 % of items), 18·6 % (*n* 555) items had one red traffic light, 12·1 % (*n* 360) with two, 7·2 % (*n* 215) with three and 0·1 % (*n* 4) with four red traffic lights (online Supplementary Table 5). When considering the number of items with at least one green traffic light (*n* 2710, 90·9 % of items), 32·9 % (*n* 980) contained one, 19·8 % (*n* 589) contained two, 23·6 % (*n* 703) contained three and 14·7 % (*n* 438) contained four green traffic lights.

**Fig. 3.**
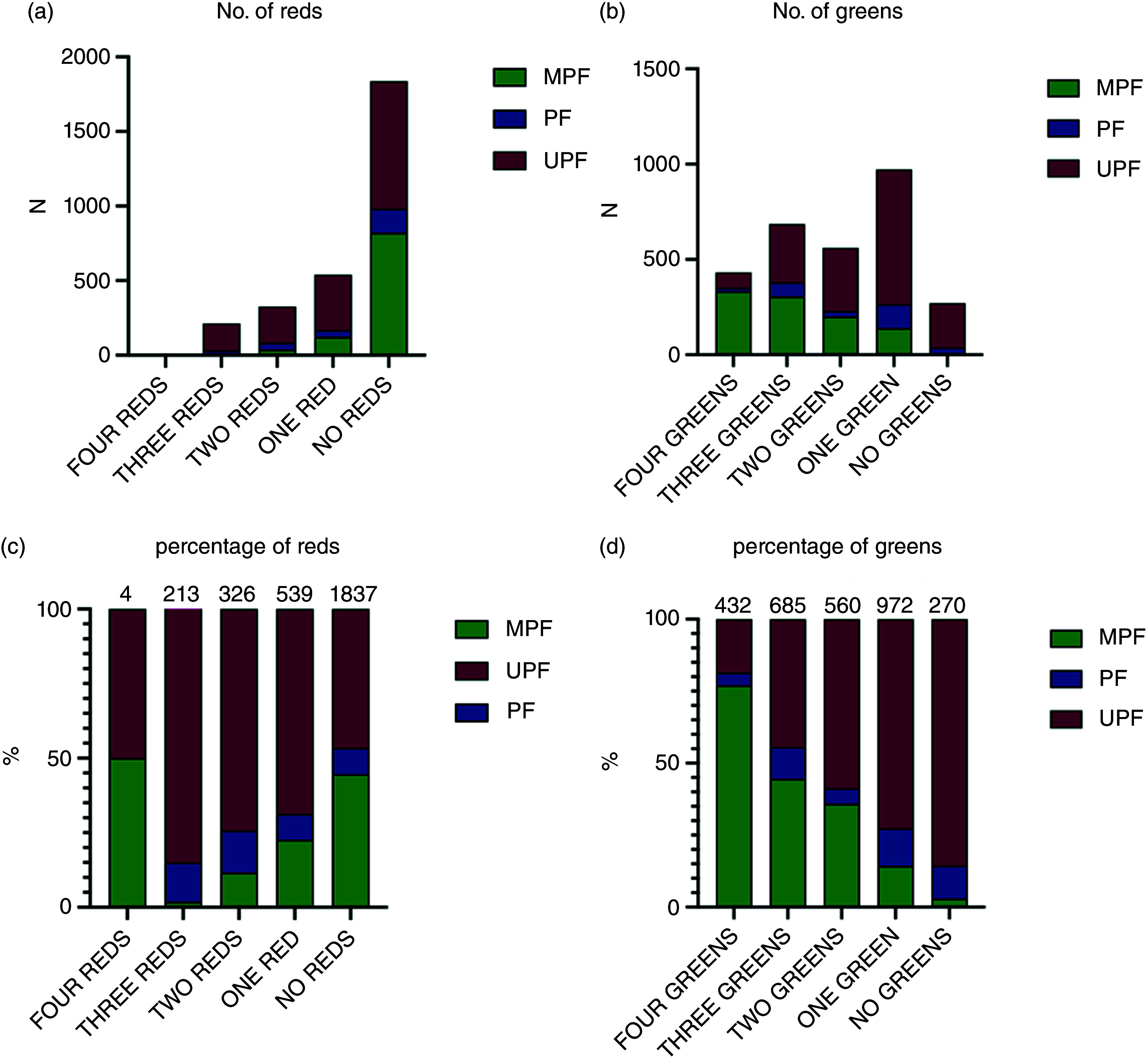
The number (a) and (b) and percentage (c) and (d) of total red (a) ad (c) or green (b) and (d) FOPL traffic lights for fat, saturated fat, total sugar and salt, stratified by NOVA group. MPF *n* 986; PF *n* 283; UPF *n* 1650. Numbers above columns in Figures 3(c) and (d) denote the number of items. FOPL, front of package label; MPF, minimally processed food; PF, processed food; UPF, ultra-processed food.

Figure 3(a)–(d) shows the number and percentage of items with none, one, two, three or four red or green FOPL traffic lights, stratified by NOVA group (processed culinary ingredients not shown) (by minimally processed and ultra-processed foods only, in online Supplementary Fig. 1(a) and (b)). The majority of items with one (67·0 %, 370 out of 555), two (67·0 %, 242 out of 360) or three (84·0 %, 181 out of 215) red traffic lights were ultra-processed foods. Nearly half of all ultra-processed foods (42·8 %, 706 out of 1650) contained one green traffic light, followed by two green traffic lights (19·9 %, 329 out of 1650), then three (18·4 %, 304 out of 1650), then zero green traffic lights (14·0 %, 231 out of 1650), with the fewest having four green traffic lights (4·8 %, 80 out of 1650). In contrast, most minimally processed foods contained four (33·8 %, 333 out of 988), then three (30·9 %, 305 out of 986), then two (20·4 %, 201 out of 986) and then one green traffic light (14·1 %, 139 out of 986).

The proportions of the number of red or green FOPL traffic lights significantly differed according to NOVA group (both *P* < 0·001) (online Supplementary Table 5). Ultra-processed foods had a higher proportion of one, two or three/four red traffic lights, whereas minimally processed foods had a lower proportion of one, two or three/four red traffic lights. Ultra-processed foods had a lower proportion of three or four green traffic lights and a higher proportion of one green or no green traffic lights, whereas minimally processed foods had a higher proportion of three or four green traffic lights and a lower proportion of one green or no green traffic lights.

Ordinal regression showed that ultra-processed foods had a significantly higher odds of containing a greater number of red FOPL traffic lights compared with minimally processed foods (*P* < 0·001, Table 4). Ultra-processed foods had a 4·84 times (95 % CI 4·00, 5·86) higher odds of containing a greater number of red FOPL traffic lights compared with minimally processed foods. For the number of green FOPL traffic lights, ultra-processed foods had a lower odds of containing a greater number of green FOPL traffic lights compared with minimally processed foods. Minimally processed foods had 7·30 times (95 % CI 6·25, 8·55) higher odds of containing a greater number of green FOPL traffic lights compared with ultra-processed foods. Processed culinary ingredients and processed foods (combined) also had a higher odds of containing a greater number of red and a lower number of green traffic lights compared with minimally processed foods (both *P* < 0·001). In sensitivity analyses, results were similar when the number of red or green FOPL traffic lights was modelled as a 5-level continuous score (online Supplementary Table 6), where ultra-processed foods had 1·25 (95 % CI –1·34, –1·16) fewer green FOPL traffic lights and 0·63 (95 % CI 0·56, 0·70) more red FOPL traffic lights.

**Table 4.** Ordinal regression modelling the association between NOVA group and the presence of an increasing number of red/green FOPL traffic lights

	Exp(B)	95 % confidence interval	*P* value
		Lower, Upper	
Red FOPL traffic lights			
UPF	4·842	4·002, 5·857	< 0·001
PF and PCI	5·461	4·212, 7·079	< 0·001
Green FOPL traffic lights			
UPF	0·137	0·117, 0·160	< 0·001
PF and PCI	0·212	0·169, 0·266	< 0·001

FOPL, front of package label; UPF, ultra-processed food; PF, processed food; PCI, processed culinary ingredient.

Reference = MPF.

Higher score indicates greater odds of having an increasing number of red/green FOPL traffic lights.

### Full front of package label multiple traffic light score

**Fig. 4.**
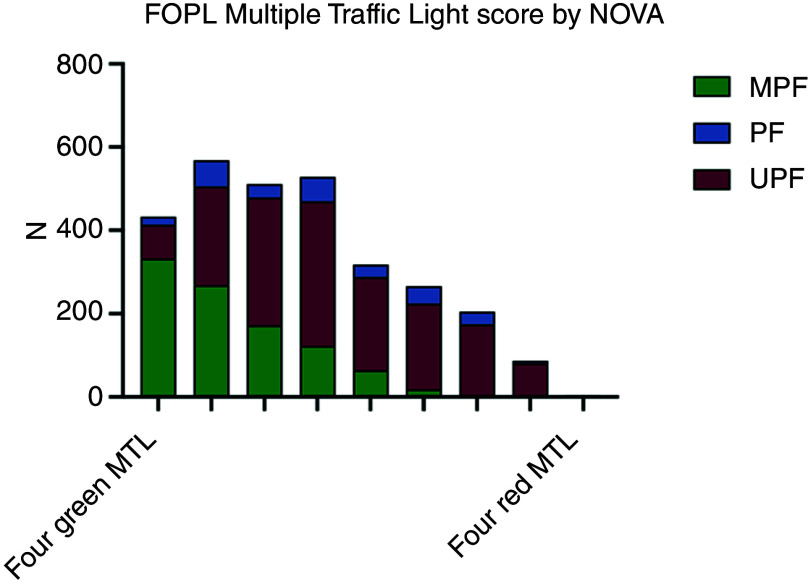
Profile of FOPL MTLs stratified by NOVA group. MPF *n* 986; PF *n* 283; UPF *n* 1650. FOPL, front of package label; MPF, minimally processed food; MTL, multiple traffic light; PF, processed food; UPF, ultra-processed food.

As a total FOPL MTL score (ranging from 4 (four green traffic lights) to 12 (four red traffic lights)), the median score was 6·0 (IQR: 5·0, 8·0), corresponding to an FOPL with either two amber and two green traffic lights or three green traffic lights and one red traffic light (AAGG or GGGR). The profile of FOPL MTL stratified by NOVA group is presented in Fig. 4 (by minimally processed and ultra-processed foods only, in online Supplementary Fig. 2). The proportions of FOPL MTL significantly differed across NOVA groups (*P* < 0·001, online Supplementary Table 7). Ordinal regression of the FOPL MTL score showed that ultra-processed foods had 7·06 times (95 % CI 6·06, 8·24) higher odds of having an unhealthier FOPL MTL score compared with minimally processed foods (*P* < 0·001, Table 5). Processed foods and processed culinary ingredients combined also had a higher odds of an unhealthier FOPL MTL score compared with minimally processed foods (OR: 5·95 (95 % CI 4·74, 7·46)). In sensitivity analyses, results were similar when the FOPL MTL score was modelled as an eight-level continuous outcome ranging from four greens to four reds/three reds and one amber (a score from 4 to 11) (online Supplementary Table 8), where ultra-processed foods had an FOPL MTL score that was 1·89 (95 % CI 1·75, 2·02) points higher than minimally processed foods (equivalent to nearly two green traffic lights replaced with two amber traffic lights, or one green traffic light replaced with one red traffic light), and when modelled as a binary outcome (above median *v*. median and below FOPL MTL score), where ultra-processed foods had 6·03 times (5·03, 7·24) higher odds of having an unhealthier FOPL MTL score (online Supplementary Table 9).

**Table 5.** Ordinal regression modelling the association between NOVA group and the presence of an increasing FOPL MTL score

	Exp(B)	95 % confidence interval	*P* value
		Lower, Upper	
UPF	7·063	6·055, 8·238	< 0·001
PF and PCI	5·947	4·742, 7·460	< 0·001

FOPL, front of package label; MTL, multiple traffic light; UPF, ultra-processed food; PF, processed food; PCI, processed culinary ingredient.

Reference = MPF.

Higher score indicates greater odds of having an unhealthier FOPL MTL score.

### Items with no red front of package label traffic light

Subgroup analyses then considered food and drink items containing no red FOPL traffic lights (*n* 1846, 61·9 %), i.e. ‘healthy’ items. 855 (46·3 %) were ultra-processed foods, 820 (44·4 %) were minimally processed foods, 162 (8·8 %) were processed foods and 9 (0·5 %) were processed culinary ingredients (online Supplementary Table 4). The most common ultra-processed foods with no red FOPL traffic lights included sandwiches (*n* 65, 7·6 %), high fibre breakfast cereals (*n* 43, 5·0 %), other milks (e.g. plant-based milk alternatives, milkshakes) (*n* 38, 4·5 %) and white bread (not high fibre, not multiseed) (*n* 35, 4·1 %) (online Supplementary Table 10).

Ultra-processed foods with no red FOPL traffic lights contained lower amounts of fat, saturated fat, total sugar and salt per 100 g than ultra-processed foods containing at least one red FOPL traffic light (all *P* < 0·001, online Supplementary Table 11). Ultra-processed foods with no red FOPL traffic lights also had a significantly lower energy density (1·52 kcal/g (IQR: 0·77, 2·43) *v*. 3·53 kcal/g (IQR: 2·51, 4·43), *P* < 0·001). There was no significant difference in protein content (6·4 g/100 g (IQR: 2·1, 10·7) *v*. 5·5 g/100 g (IQR: 3·1, 9·2), *P* = 0·761), or fibre content (*P* = 0·435), between ultra-processed foods with or without a red FOPL traffic light.

Compared with minimally processed foods with no red FOPL traffic lights, ultra-processed foods with no red FOPL traffic lights contained greater quantities of fat, saturated fat, total sugar and salt (*P* < 0·001) per 100 g (online Supplementary Table 12). Ultra-processed foods had a significantly higher energy density than minimally processed foods (1·52 kcal/g (IQR: 0·77, 2·43) *v*. 0·75 kcal/g (IQR: 0·32, 1·34), *P* < 0·001) and processed foods (1·08 kcal/g (IQR: 0·59, 1·75) *P* < 0·001). There was no significant difference in protein (*P* = 0·184) or fibre (*P* = 0·231) content between minimally processed foods and ultra-processed foods with no red FOPL traffic lights, but the water content of ultra-processed foods was significantly lower than that of minimally processed foods (*P* < 0·001). Processed foods with no red FOPL traffic lights also contained significantly more saturated fat, total sugar, salt and energy than minimally processed foods with no red FOPL traffic lights. In sensitivity analyses, binary regressions with median cut-off showed similar associations between NOVA groups and nutrient content (online Supplementary Table 13). Sensitivity analyses further considered items with no red FOPL traffic lights and two or more green FOPL traffic lights (*n* 1403, 47·1 %) (corresponding to an item with a median or lower FOPL MTL score with no red FOPL traffic lights). 738 (52·6 %) were minimally processed foods, 554 (39·5 %) were ultra-processed foods, 102 (7·3 %) were processed foods and 9 (0·6 %) were processed culinary ingredients. Ultra-processed foods with no reds and at least two greens had lower fat, saturated fat, total sugar and salt content per 100 g than ultra-processed foods with reds or less than two green FOPL traffic lights (all *P* < 0·001) (online Supplementary Table 14). Ultra-processed foods with no reds and at least two greens contained greater quantities of fat, saturated fat, total sugar and salt than minimally processed foods with no reds and at least two green FOPL traffic lights (*P* < 0·001) and had a significantly higher energy density (1·07 kcal/g (IQR: 0·48, 2·21) *v*. 0·71 kcal/g (IQR: 0·29, 1·24), *P* < 0·001) and processed foods (0·74 kcal/g (IQR: 0·40, 1·02), *P* < 0·001) (online Supplementary Table 15).

### Hyper-palatable food

**Fig. 5.**
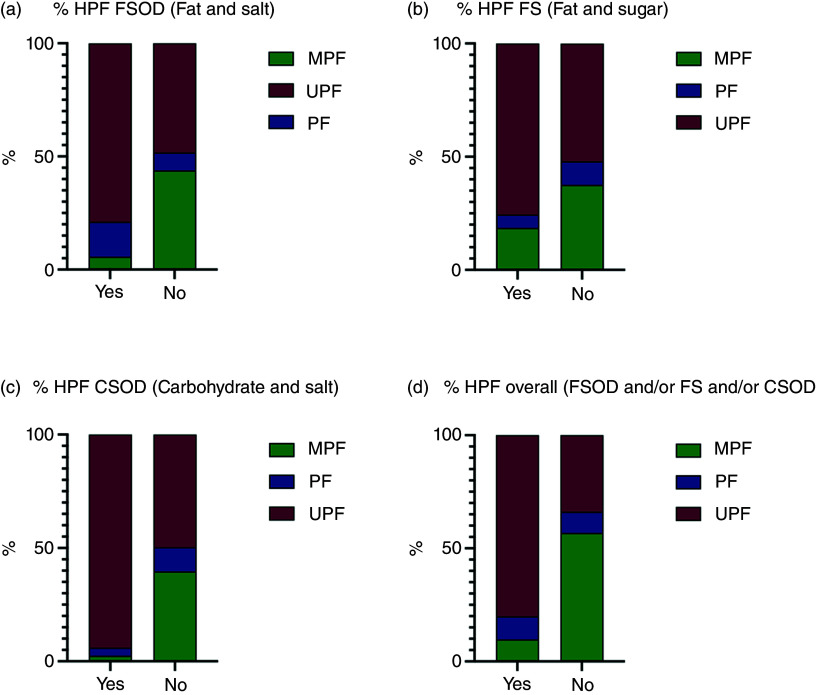
Proportions of food items meeting criteria for hyper-palatability based on: (1) fat and Na (a) (2) fat and simple sugars (b) (3) carbohydrates and Na (or any of the three clusters (d) (processed culinary ingredients not shown). (1) fat and Na (> 25 % kcal from fat, ≥ 0·30 % Na content by weight) hyperpalatable *n* 642; (2) fat and simple sugars (> 20 % kcal from fat, > 20 % kcal from sugar) hyperpalatable *n* 421; (3) carbohydrates and Na (> 40 % kcal from carbohydrates, ≥ 0·20 % Na by weight) hyperpalatable *n* 365; any of the three clusters hyperpalatable *n* 1246. CSOD, carbohydrate and salt; FOPL, front of package label; FS, fat and sugar; FSOD, fat and salt; HPF, hyper-palatable food; MPF, minimally processed food; MTL, multiple traffic light; PF, processed food; UPF, ultra-processed food.

When stratified by hyper-palatability, 46·8 % (*n* 1246) of all food items (*n* 2665) were classified as being hyper-palatable based on their fat, Na, sugar and carbohydrate content. Of which, 79·8 % were ultra-processed foods (994 out of 1246). Across each cluster, the majority of HPF defined by (1) fat and Na (78·5 %, 504 out of 642), (2) fat and simple sugars (75·5 %, 318 out of 421) or (3) carbohydrates and Na (93·7 %, 342 out of 365) were ultra-processed foods (Fig. 5). Across all items, a significantly greater proportion of ultra-processed foods than minimally processed foods were classed as HPF for: (1) fat and Na, (2) fat and simple sugars or (3) carbohydrates and Na and overall (combining all clusters) (all *P* < 0·001) (online Supplementary Table 16). When considering ‘healthy’ food items (i.e. with no red FOPL traffic lights, *n* 1620), there was still a significantly greater proportion of ultra-processed foods than minimally processed foods that were defined as HPF, based on: (1) fat and Na and (3) carbohydrates and Na and overall (all *P* < 0·001), but there was no significant difference for (2) fat and simple sugars (*P* = 0·367).

## Discussion

The findings from this analysis indicate that ultra-processed foods tend to have an unhealthier nutritional profile than minimally processed foods, but not processed foods. Ultra-processed foods contained greater amounts of fat, saturated fat, total sugar and salt than minimally processed foods, were more energy dense and were more likely to be classed as hyper-palatable. Compared with processed foods, ultra-processed foods contained similar amounts of fat, saturated fat and salt, but greater amounts of sugar and were more energy dense. Ultra-processed foods were more likely to have fewer green FOPL traffic lights, a greater number of red FOPL traffic lights and be rated as unhealthier based on their overall FOPL MTL score. However, not all ultra-processed foods had an unhealthy nutrient profile. Over half of ultra-processed foods had no red FOPL traffic lights, and a significant number of ultra-processed foods had a FOPL MTL score similar to minimally processed foods, with nearly half of items with no red FOPL traffic lights being classed as ultra-processed foods. However, ultra-processed foods with no red FOPL traffic lights still had a worse nutritional profile and higher energy density than minimally processed foods with an equivalent FOPL MTL score and were still more likely to be classed as hyper-palatable, with greater combinations of fat and/or sugar/starch and or/salt content. These results suggest that the FOPL MTL system does not fully differentiate between ultra-processed foods and minimally processed foods, only partially capturing the extent and purpose of food processing.

Aspects of ultra-processing, such as changes to the food matrix, greater energy density and the combination of nutrients not usually found in minimally processed foods, have been suggested to alter oro-sensory exposure time, increasing eating rates and resulting in overconsumption^(18,20,36)^. In this study, ultra-processed foods were more energy dense than minimally processed foods and processed foods. Even when considering only ‘healthy’ items with no red FOPL traffic lights, ultra-processed foods still had double the energy density of minimally processed foods (1·52 kcal/g *v*. 0·75 kcal/g). Lowering energy density can lower daily energy intake in a strong and linear fashion^(37)^, with an average 223 kcal reduction in energy intake when lowering meal energy density from 1·5 kcal/g to 1·1 kcal/g^(38)^. In a metabolic ward crossover study comparing a 2-week ultra-processed diet (1·36 kcal/g) *v*. a 2-week minimally processed diet (1·09 kcal/g) matched for presented energy and macronutrients, participants consumed ∼500 kcal per day more on the ultra-processed than minimally processed diet, resulting in 0·9 kg weight gain on the ultra-processed diet, but 0·9 kg of weight loss on the minimally processed diet^(16)^. Hyper-palatability has also been suggested to be important for food choice and consumption^(35)^. Many ultra-processed foods have hyper-palatable properties, such as combinations of fat and sugar not usually seen in nature, which have been proposed to have addictive-like qualities by inducing a greater hedonic response when consumed, increasing reward-driven eating^(20,35,39)^. In the metabolic ward study, both energy density (45·1 ± 13·6 %) and hyper-palatability (41·9 ± 6·5 %) explained large proportions of the greater daily non-beverage energy intake on the ultra-processed *v*. minimally processed diet^(40)^. In addition, ultra-processed foods have also been described by their extensive matrix degradation, which can make them softer and easier to consume at a faster rate^(36)^. Therefore, ultra-processed foods may capture several characteristics that may predispose to overconsumption that are not sufficiently reflected in current FOPL guidance. These findings suggest that within the UK food and drink supply, choosing healthier ultra-processed foods based on the FOPL MTL score may still predispose to increased energy intake, compared with minimally processed foods with a similar FOPL MTL score.

The NOVA classification does not explicitly differentiate food and drink based on their nutrient content. But, ultra-processed diets tend to be nutritionally poorer^(13)^, evident by the partial overlap with the FOPL MTL score. However, although ultra-processed foods tend to have an unhealthier nutritional profile than minimally processed foods, not all do. This finding is in line with studies from other countries using different nutrient indices^(23,28)^. For example, comparing breakfast cereals across NOVA groups indicated that minimally processed foods were not always healthier based on their nutritional profile, with some ultra-processed foods also scoring well on NutriScore and differences being dependent on the serving size used for comparison^(41)^. Given the adverse impacts of high intakes of nutritionally poor foods high in fat, sugar and salt^(42–44)^, transnational corporations have reformulated their products to contain less fat, saturated fat, added sugar or salt^(45)^. Previous analyses based on nutrient profiling have suggested that ultra-processed foods with better nutritional profiles may be considered healthy^(46)^, particularly if they carry a nutrient or health claim. Notably, a large proportion of ultra-processed foods in this study contained one green FOPL traffic light, such as low-fat ready meals, sauces and puddings, which could carry nutrient claims. Recently, Hess et al. designed an ultra-processed diet meeting the USA Dietary Guidelines for Americans, scoring highly on the Healthy Eating Index^(47)^. Similarly in this analysis, a number of ultra-processed food groups have a healthier FOPL MTL score, suggesting that individuals in the UK may also be able to follow public health dietary guidance with a high ultra-processed food intake. However, it is unclear whether this reflects a healthy diet, or instead indicates a fundamental flaw in nutrient-based approaches^(48)^. Thus, to what extent a diet high in ultra-processed food, but consisting of items with better nutritional quality and containing nutrition/health claims can constitute a healthy diet remains to be seen.

Whether the inclusion of the NOVA classification and avoidance of ultra-processed foods should be recommended in dietary guidelines is currently debated^(49)^. The Scientific Advisory Committee on Nutrition report on processed foods concluded that there is insufficient evidence to warrant inclusion of food processing into dietary guidelines, given the possibility that ultra-processing is already covered by existing dietary recommendations^(10)^. The results here highlight that FOPL MTL scores carry important information not captured by the NOVA classification. Exclusively using the NOVA classification to choose food and drinks might lack the granularity to identify the nutrient quality of items, excluding potentially healthy food choices unnecessarily. However, the most up-to-date scientific guidance from the American Heart Association and American Society for Preventive Cardiology includes advice to choose minimally processed foods and/or minimise ultra-processed food intake, alongside standard dietary guidance to limit foods high in fat, sugar and salt^(2,3)^. Such recommendations indicate that consumers should consider not just the nutrient content but also the processing of their food purchases. FOPL encourage healthier in-supermarket food purchases^(50)^, and previous studies suggest Nutri-Score to be most effective in improving consumer understanding of the healthfulness of food products in the UK and in Europe^(9,51,52)^. Current FOPL in the UK use reductive approaches to provide consumers with guidance regarding which products to consume more of and those to consume less of. FOPL do not take into account the extent and purpose of food processing and may be insufficient to help inform consumers to choose minimally processed foods over ultra-processed foods. In addition, processed foods and ultra-processed foods had similar nutrient content and would be expected to carry similar FOPL MTL. But, ultra-processed foods were still more energy dense, potentially due to the significantly lower water content of ultra-processed foods. It is therefore important to consider whether current FOPL provide adequate information to guide consumers towards making healthy in-store food choices. Further clinical evidence examining the role of food processing independent of current dietary guidance will be important prior to changes to food labelling within the retail environment. There is also the potential that the FOPL nutrient cut-offs could be a limitation. To date, there is limited evidence regarding optimal cut-off points for HFSS FOPL, or portion sizes. Whether altered cut-off points would have the potential to better differentiate between NOVA groups remains unclear.

Therefore, a combined approach to dietary guidelines and food labelling that considers both the extent and purpose processing and existing indicators of dietary quality may provide the most informative guidance^(53)^. How best to communicate this combined guidance needs to be deliberated, to avoid presenting a complex public health message and stigmatising choosing ultra-processed foods, often being the only option for some individuals. Ultra-processed foods tend to be cheaper than minimally processed foods^(22,54)^. In the UK, a lower social class is associated with greater ultra-processed food intake^(55)^ and income with poorer diet quality^(56)^. Policy makers must therefore consider the financial implications and abilities of the UK public to shift towards a minimally processed diet and away from ultra-processed foods, particularly with the current cost of living crisis^(57)^, and issues relating to accessibility of minimally processed foods.

### Strengths and limitations

There are several strengths of this study, including the large UK database of nationally representative food and drink items, with a matching nutrient database containing average nutrient compositions. Products were compared not only on their nutrient content but also on their FOPL coding used at consumer point of purchase and wider characteristics that influence food intake. Limitations include the analysis per 100 g, which although allows for comparability between food and drink items, does not reflect nutrient intakes of actual portions that may be consumed. Despite the NOVA classification being the most widely used and most accessible method to identify between types of food processing, there have been criticisms over its use and operationalisability^(58)^. Issues such as applying the classification^(59)^, or low inter-rater consistency in coding^(60)^, have been suggested. Despite agreement amongst the authors on classification of items in this study, a small number of items could have been coded into more than one group. However, other studies indicate that only 5–10 % of items are potentially misclassified or ambiguous, with the vast majority of items consistently coded into the same NOVA group by different coders^(61–63)^. In addition, Scientific Advisory Committee on Nutrition outlined that NOVA, despite its limitations, was the only processing classification meeting all five of their screening criteria, including being applicable to the UK population^(51)^. The HPF index was defined from a systematic review of definitions and has only been developed for food and not drink, which limits the wider application of these findings. It was also not possible to assess other properties of items that may influence eating rate and energy intake in this analysis, such as textural properties^(36)^.

### Conclusion

The NOVA classification partially overlaps with the nutrient content of UK food and drink items. Ultra-processed foods tend to have an unhealthier nutritional profile according to FOPL and a higher energy density than minimally processed foods, with greater red FOPL traffic lights and fewer green FOPL traffic lights. Ultra-processed foods were nutritionally similar to processed foods, but more energy dense. However, many ultra-processed foods contained no red FOPL traffic lights and multiple green FOPL traffic lights, with a nutritional profile equivalent to many minimally processed foods. However, ‘healthy’ ultra-processed foods still tended to contain more fat, saturated fat, total sugar and salt than minimally processed foods with an equivalent FOPL traffic light score and were more energy dense and more likely to be hyper-palatable. With not all ultra-processed items fitting within the HFSS categories, and some appearing healthy according to FOPL MTL, there appears to be a lack of clarity around how processing might be used in line with the UK dietary recommendations and on FOPL. These results have important implications for understanding how consumers may interpret the relative healthiness of minimally processed foods and ultra-processed foods and for updating UK food and drink labelling.

## Supporting information

Dicken et al. supplementary material Dicken et al. supplementary material

## Supplementary material

For supplementary material/s referred to in this article, please visit https://doi.org/10.1017/S0007114524000096
